# Cronkhite-Canada Syndrome: A Case Report

**DOI:** 10.31729/jnma.7407

**Published:** 2022-05-31

**Authors:** Prakash Sapkota, Ram Bahadur Gurung, Ashish Shrestha, Isha Paudel, Pramita Shrestha

**Affiliations:** 1 Department of Internal Medicine, Dhulikhel Hospital, Kathmandu University School of Medical Sciences, Dhulikhel, Kavre, Nepal; 2 Department of Public Health and Community Programs, Dhulikhel Hospital, Kathmandu University School of Medical Sciences, Dhulikhel, Kavre, Nepal

**Keywords:** *alopecia*, *case reports*, *cronkhite-canada syndrome*, *hyperpigmentation*

## Abstract

Cronkhite-Canada Syndrome is a rare disease characterised by diffuse gastrointestinal polyposis, abdominal pain, diarrhoea, cutaneous and mucosal hyperpigmentation, alopecia, and onychodystrophy. Here we report a case of a 40-year-old female with Cronkhite-Canada Syndrome, who presented with the complaints of diffuse abdominal pain, blood mixed stools, and diarrhoea associated with tenesmus. She had nausea and reduced appetite and lost 10 kgs in 3 months. She had hair fall (alopecia), atrophic changes of nails (onychodystrophy), and hyperpigmentation of the skin in fingers, tongues, and lips. Histopathological biopsy of the gastric and colonic biopsy revealed polypoid edematous mucosa and the colonic biopsies showed scattered dilated glands with inflammatory exudate and mucin. She got *Entamoeba histolytica* and COVID-19. She received respective antibiotics and protein diets that helped relieve the symptoms. After 4 weeks of steroids, her symptoms improved drastically. Corticosteroids, treating co-infection along with nutritional counselling can be helpful to relieve the symptoms.

## INTRODUCTION

Cronkhite-Canada Syndrome (CCS) is sporadic, predominantly in Japan and United States with higher than 500 cases globally in half a century.^1^ CCS is characterised by abdominal pain, diarrhoea, diffuse polyposis of the gastrointestinal tract and ectodermal changes (hyperpigmentation, alopecia, onychodystrophy).^2^ It is a non-hereditary disorder of unidentified aetiology with a poor prognosis with more than half of the reported mortality of the cases within 5 years. However, treatment options are available.^3^ Here we report a case of a 40-year-old female with Cronkhite-Canada Syndrome, managed successfully with antibiotics, steroids and nutritional therapy.

## CASE REPORT

A 40-year-old female presented in Dhulikhel Hospital, Nepal with complaints of diffuse abdominal pain and blood mixed stools. Her abdominal pain heightened with the food intake. She also complained of diarrhoea associated with tenesmus. Her stools were foulsmelling and intermittently watery or soft, initially black coloured and later with fresh blood. Since the onset of the symptoms, her stool frequency has increased up to 20 times per day. She had nausea and reduced appetite and lost 10 kgs in 3 months. The case had hair fall (alopecia), atrophic changes of nails (onychodystrophy), and hyperpigmentation of the skin in fingers, tongues, and lips. However, she did not have joint pain, frothy urine, cough, chest pain, palpitations, or fever. There was no significant family history.

**Figure 1:**
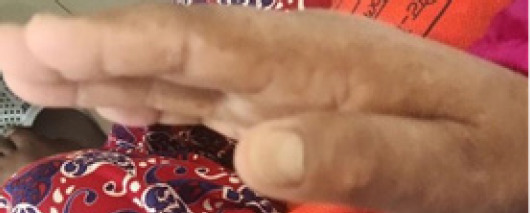
Hyperpigmentation of fingers.

She was a known case of pulmonary hypertension, hypothyroidism, and systemic hypertension (under carvedilol and losartan medication). On physical examination, her pulse rate was 95 beats per minute, her blood pressure was 140/90 mmHg and was afebrile with a normal respiratory rate and her measured body-mass index (kg/m^2^) was 26.67. The patient had hyperpigmentation of fingers (Figure 1).

**Figure 2:**
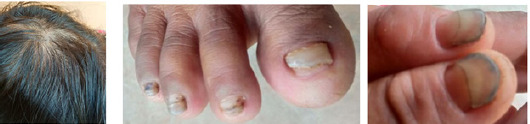
Hair loss (alopecia) and onychodystrophy (dystrophic changes in nails).

The patient also had alopecia and when a hair pull test was done, it showed that more than 10 hairs were picked out. There were also black coloured tongue, dark-coloured lips and onychodystrophy/dystrophic changes of nails of all four limbs (Figure 2).

The author obtained informed written consent from the case, assured voluntary participation, and maintained confidentiality and privacy throughout the study. Her stool microscopic routine and examination revealed cysts of *Entamoeba histolytica,* 10 to 12 white blood cells, and 40 to 45 occult red blood cells (Table 1).

**Table 1:** Laboratory findings.

Parameters	Findings	Remarks
Total count (ul)	6900	Normal
Differential count (%)	N 54, L 36	Normal
Hemoglobin (g/dl)	13	Normal
Erythrocyte sedimentation rate (mm/hrs)	23	Normal
C-reactive protein (mg/l)	7	Normal
Prothrombin time/ International normalization ratio (seconds)	13/1.0	Normal
Random blood sugar (mg/dl)	76	Normal
Sodium (meq/l)	141	Normal
Potassium (meq/l)	4.6	Normal
Urea (mg/dl)	8	Normal
Creatinine (mg/dl)	0.4	Normal
Rheumatoid factor (IU/ml)	Negative	Normal
Anti-nuclear antibody	Negative	Normal

**Figure 3:**
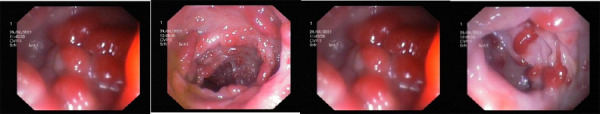
Multiple sessile polyps seen during colonoscopy.

Gastroscopy revealed diffusely erythematous raised lesions with islands of normal-looking mucosae in between noted throughout the stomach (more in the corpus) along with similar congested mucosal lesions in the duodenum, with a normal oesophagus. Colonoscopy showed findings suspicious of polyposis syndrome in the whole colon with mainly sessile polyps throughout the whole colon, larger ones more distally (Figure 3).

Histopathological biopsy of the gastric and colonic biopsy revealed polypoid edematous mucosa, mild chronic lymphoplasmacytic inflammation and mild reactive changes. In the colon as well as stomach, the oedema was present more in the superficial mucosal aspect. The deeper glands did not appear to be involved. The colonic biopsies showed scattered dilated glands with inflammatory exudate and mucin. No surface ulcerations or adenomas were found. However, her stool investigation revealed *Entamoeba histolytica* and she also got infected with COVID-19 with mild symptoms, which led to a short delay in diagnosis and treatment. Hence, to avoid this delay, clinicians should consider gastrointestinal manifestations as well as ectodermal changes to come to a diagnosis.

The clinical, gastroscopic, colonoscopy and histopathological findings led to the clear diagnosis of Cronkhite-Canada Syndrome. Since the patient was found to be positive for *Entamoeba histolytica,* initial treatment with oral ciprofloxacin 500 mg twice a day and oral metronidazole 400 mg thrice a day was given for 7 days. A recommendation was made to increase the amount of protein in the diet. This helped in the transient relief of symptoms. After 2 weeks of subsidence from this treatment, the patient was counselled well. Oral prednisolone 60 mg once daily was started and tapered accordingly. A high protein diet was continued.

Following the treatment, after 4 weeks of the first diagnosis, the patient was symptomatically improved with relief from the abdominal pain and diarrhoea, regaining of hair, better nails, increased appetite, absence of blood in stool, and weight gain of 7 kg. Echocardiography was done which showed normal pulmonary arterial pressure. Her blood pressure was within normal range so antihypertensive was tapered accordingly.

Following 14 weeks of serially tapered steroid therapy, the patient underwent a follow-up colonoscopy which revealed regression of previous multiple polyps. One bleeding sessile polyp which was noted in the colon was removed by snare after saline lifting. One large polyp was found in the descending colon and a few small polyps in the sigmoid. Other than this the colonoscopy exhibited normal mucosa. Her repeat biopsy report showed negative for dysplasia and malignancy. The patient was symptomatically better with gradual relief from the abdominal pain and diarrhoea to a great extent, regaining of hair, better nails, increased appetite, absence of blood in stool, and weight gain of 10 kgs.

**Figure 4:**
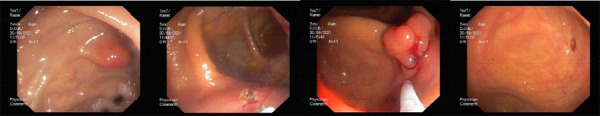
The progressive follow-up colonoscopy reports.

## DISCUSSION

With only over 500 cases being reported worldwide, Cronkhite-Canada Syndrome (CCS) has been considered a sporadic and rare clinical condition.^4^ Clinical description of CCS was first made by Cronkhite and Canada in 1955.^5^ Its occurrence is reported to be sporadic with no strong evidence to suggest a hereditary predisposition^4^ except one reported in father and son of Indian descent.^6^ The case in this study also did not report any hereditary history. Patients of European and Asian descent are affected by more^4^ than 75% of cases from Japan.^6^ As per our knowledge, the case presented in this study can be considered the first few cases in Nepal. A study of 210 patients in Japan found that the age of onset of symptomatic CCS ranged from 31 to 86 years, with an average age of 63.5 years.^3^ The age of the female case presented in this study was 40 years. A study in Japan reflected slightly more cases among males than females with the ratio of males to females being 1.84:1,^3^ whereas a study conducted in China illustrated that the ratio of males to females is 2:1.^7^

The aetiology of CCS is under scrutiny. The evidence of elevated levels of autoimmune markers like antinuclear antibodies and anti-neutrophil cytoplasmic antibodies advise autoimmune as one of the etiologies.^8^ However, some studies have reported the presence of few cases with normal levels of these markers and the case in this study is one of such.^9^ Also, this case had comorbidity of hypothyroidism and pulmonary hypertension. Some studies have depicted an association of CCS with hypothyroidism and various autoimmune originated diseases such as systemic lupus erythematosus, rheumatoid arthritis, and scleroderma.

Although the symptoms may vary, CCS typically presents with gastrointestinal polyposis and ectodermal changes which are also known as pigmentation-alopecia-onychatrophia syndrome.^6^ A study reported that clinical manifestations of CCS can be divided into five types based on initial symptoms: i) diarrhoea; ii) taste abnormalities; iii) dry mouth; iv) hair loss and nail atrophy; and v) loss of appetite, malaise, nail atrophy, hair loss, and dysgeusia in the absence of diarrhoea.^4^ The case in the study reported few consistent symptoms such as diarrhoea and ectodermal changes. Gastrointestinal polyposis is closely related to the malabsorption which induced these ectodermal changes as well as diarrhoea.^10^

The past studies state that a few patients have been successfully treated with antibiotics, though usually as a part of a multi-drug regimen. Co-existing infections have occurred in a few patients, raising the possibility of associated immunodeficiency. A case of CCS has been reported of remission after treatment with an *anti-Helicobacter pylori* regimen.^11^ The index case also showed transient remission of symptoms after treatment with antibiotics for *Entamoeba histolytica.*

Gastrointestinal polyps in the cases of CCS are retention type or hamartomatous in nature distributed throughout the stomach and colon (90%), small bowel (80%), and rectum (67%) with characteristic oesophagal sparing.^11^ The case of this study has consistent findings with that of a few other studies whose histopathological reviews of biopsies obtained from these polyps revealed that these polyps are like that of juvenile, adenomatous polyps, or inflammatory type polyps, however, they were additionally marked by striking stromal and lamina propria edematous changes, eosinophilic inflammation was cystically dilated and had distorted glands with inflammatory infiltration.^3^ CCS is differentiated from other hamartomatous polyposis syndromes by its widespread polyp distribution in the stomach, small bowel, and colon and sparing of the oesophagus.

Several medical therapies have been used in patients with CCS. Nutritional support, antibiotics, corticosteroids, anabolic steroids, and histamine-receptor antagonists have all been used with varying degrees of success.^11^ A study revealed that appropriate medical therapy can alter the natural history of CCS.^4^ Oral corticosteroid therapy (30-49 mg/day) appeared to be effective for active CCS. Its benefits, including clinical improvement and polyp regression, are usually apparent within 12 months. The index case of this study unveiled significant improvement after receiving the treatment with corticosteroids.

The prognosis of CCS is poor, with a 5-years mortality rate of 55% and most of the mortality being associated with malnutrition, hypoalbuminemia, repetitive infection, sepsis, heart failure and gastrointestinal bleeding.^2^ Increased mortality rate is attributed to late clinical intervention.^1,3^ Delays in diagnosis are common, primarily due to the non-familiarity of physicians with this rare entity, resulting in a poor outcome.^11^ The index case initially presented with abdominal pain and tenesmus associated with diarrhoea and stool investigation revealed *Entamoeba histolytica,* infected with COVID-19 with mild symptoms which led to a short delay in diagnosis and treatment. Hence, to avoid this delay, clinicians should consider gastrointestinal manifestations as well as ectodermal changes before coming to a diagnosis.

The strength of the study is that the case was clinically investigated in detail and followed up for about 4 months. The case received several possible treatment support to get healed from the symptoms timely. Since the case was limited to single, this could have been conducted in various age groups to understand the varieties of the case condition.

The aetiology of this rare disease condition is still under investigation. The study highlights the presence of comorbidities such as hypothyroidism, systemic hypertension, and pulmonary hypertension that may have been associated with the CCS. The features of abdominal pain, diarrhoea, onychodystrophy mucosal hyperpigmentation alopecia and gastrointestinal polyposis should be well explored and understood to further intervene against the CCS. Corticosteroids, treating co-infection along with nutritional counselling can be helpful to relieve the symptoms. Moreover, further studies should be conducted with a greater sample size to obtain a better understanding of findings on the symptoms, aetiology, diagnosis, and treatment of CCS.

## References

[1] Yoshimoto T, Okamoto T, Fukuda K (2021). Cronkhite-Canada Syndrome: A Rare COVID-19 Mimicker.. Am J Gastroenterol..

[2] Yun SH, Cho JW, Kim JW, Kim JK, Park MS, Lee NE (2013). Cronkhite-Canada syndrome associated with serrated adenoma and malignant polyp: a case report and a literature review of 13 Cronkhite-Canada syndrome cases in Korea.. Clin Endosc..

[3] Watanabe C, Komoto S, Tomita K, Hokari R, Tanaka M, Hirata I (2016). Endoscopic and clinical evaluation of treatment and prognosis of Cronkhite-Canada syndrome: a Japanese nationwide survey.. J Gastroenterol..

[4] Murata K, Sato K, Okada S, Suto D, Otake T, Kohgo Y (2020). Cronkhite-Canada syndrome successfully treated by corti costeroids before presenting typical ectodermal symptoms.. Case Rep Gastroenterol..

[5] Cronkhite LWJ, Canada WJ (1955). Generalized gastrointestinal polyposis; an unusual syndrome of polyposis, pigmentation, alopecia and onychotrophia.. N Engl J Med..

[6] Patil V, Patil LS, Jakareddy R, Verma A, Gupta AB (2013). Cronkhite-Canada syndrome: a report of two familial cases.. Indian J Gastroenterol..

[7] She Q, Jiang JX, Si XM, Tian XY, Shi RH, Zhang GX (2013). A severe course of Cronkhite-Canada syndrome and the review of clinical features and therapy in 49 Chinese patients.. Turk J Gastroenterol..

[8] Ota S, Kasahara A, Tada S, Tanaka T, Umena S, Fukatsu H (2015). Cronkhite-Canada syndrome showing elevated levels of antinuclear and anticentromere antibody.. Clin J Gastroenterol..

[9] Wang J, Zhao L, Ma N, Che J, Li H, Cao B (2017). Cronkhite-Canada syndrome associated with colon cancer metastatic to liver: A case report.. Medicine (Baltimore)..

[10] Seshadri D, Karagiorgos N, Hyser MJ (2012). A case of cronkhite-Canada syndrome and a review of gastrointestinal polyposis syndromes.. Gastroenterol Hepatol (N Y)..

[11] Fan RY, Wang XW, Xue LJ, An R, Sheng JQ (2016). Cronkhite-Canada syndrome polyps infiltrated with IgG4-positive plasma cells.. World J Clin Cases..

[12] Iqbal U, Chaudhary A, Karim MA, Anwar H, Merrell N (2017). Cronkhite-Canada Syndrome: A Rare Cause of Chronic Diarrhea.. Gastroenterology Res..

